# Children Change Their Answers in Response to Neutral Follow‐Up Questions by a Knowledgeable Asker

**DOI:** 10.1111/cogs.12811

**Published:** 2020-01-06

**Authors:** Elizabeth Bonawitz, Patrick Shafto, Yue Yu, Aaron Gonzalez, Sophie Bridgers

**Affiliations:** ^1^ Department of Psychology Rutgers University – Newark; ^2^ Department of Mathematics and Computer Science Rutgers University – Newark; ^3^ Office of Education Research National Institute of Education Singapore; ^4^ Now at Google; ^5^ Department of Psychology Stanford University

**Keywords:** Cognitive development, Social inference, Bayesian model

## Abstract

Burgeoning evidence suggests that when children observe data, they use knowledge of the demonstrator's intent to augment learning. We propose that the effects of social learning may go beyond cases where children observe data, to cases where they receive no new information at all. We present a model of how simply asking a question a second time may lead to belief revision, when the questioner is expected to know the correct answer. We provide an analysis of the CHILDES corpus to show that these neutral follow‐up questions are used in parent–child conversations. We then present three experiments investigating 4‐ and 5‐year‐old children's reactions to neutral follow‐up questions posed by ignorant or knowledgeable questioners. Children were more likely to change their answers in response to a neutral follow‐up question from a knowledgeable questioner than an ignorant one. We discuss the implications of these results in the context of common practices in legal, educational, and experimental psychological settings.

## 1 Introduction

How do children form their beliefs about the world given that their experiences are so sparse, noisy, ambiguous, and often indirect? Considerable evidence suggests that children can learn from their own interventions (Bonawitz et al., 2012; Cook, Goodman, & Schulz, 2011; Schulz, Gopnik, & Glymour, 2007; Sodian et al., 1991), from patterns of covariation (Gweon & Schulz, 2011; Kushnir & Gopnik, 2007; Schulz, Bonawitz, & Griffiths, 2007; Schulz & Gopnik, 2004), and from testimony of others (e.g., Koenig & Harris, 2005). Based on each of these sources, children may modify their beliefs on the basis of evidence. However, recent research suggests that in social situations children go “beyond the evidence”—they leverage the knowledge and intent of demonstrators to strengthen inferences about the world (Bonawitz et al., 2011; Buchsbaum et al., 2011; Butler & Markman, 2012; Gweon, Tenenbaum, & Schulz, 2010; Horowitz & Frank, 2016; Kushnir, Xu, & Wellman, 2010). For example, Bonawitz et al. (2011) found that children spontaneously use the inferred goals and knowledge state of an adult (e.g., who knowledgeably taught about a function of a toy vs. accidentally demonstrated it) to guide their exploration and learning. Children who observed the knowledgeable and helpful demonstrator explored the toy less than children who observed the naïve demonstrator, suggesting that children used the demonstrator's knowledge and intent to augment inferences based on data and update their beliefs about the world.

We propose that children's social inferences may be so prevalent that they affect children's interpretation of even seemingly innocuous events. For example, early work by Dale, Loftus, and Rathbum (1978) has demonstrated that ambiguous but leading questions such as “Did you see any …?” leads preschool‐aged children to misremember positively; this work has inspired a huge research program on children's susceptibility to pragmatics in testimony. However, even after providing initially correct reports, considerable research has shown that, in response to neutral follow‐up questions, children tend to change their responses. For example, Poole and White (1991) found that, in contrast to adults, 4‐year‐olds were more likely to change their responses to repeated “yes or no” questions (see also Poole & White, 1993, 1995). More recently, Krahenbuhl, Blades, and Eiser (2009) found that children as young as 4 years changed over a quarter of their responses to repeated questions, resulting in a decline in accuracy. In Howie, Sheehan, Mojarrad, and Wrzesinska (2004), 4‐year‐old children were more likely than older children to change responses toward an incorrect answer on repeated questioning. Furthermore, they found that providing rationale for the repeated questioning from a single interviewer reduced these younger children's shifting.

Taken together, this work demonstrates that by simply asking children the same question a second time, without providing additional evidence, children are very likely to switch their predictions. This tendency for children to switch responses after seemingly innocuous follow‐up questions might be interpreted as an irrational behavior: Children may have limited memory for previous responses; they may fail to understand the question; or they may otherwise be behaving “noisily.” However, the tendency to switch responses following neutral questions might instead be indicative of a rational process: Children might be making inferences about the intentions of the questioner and using these social inferences to reinterpret the validity of their own beliefs.

There is good reason to believe that children carefully consider others' belief states when reasoning. First, there is a rich literature demonstrating that even preschool‐aged children are able to reason about a teacher's knowledge state to decide whether to trust information (Clément, Koenig, & Harris, 2004; Harris & Corriveau, 2011; Jaswal & Neely, 2006; Koenig & Harris, 2005; Pasquini, Corriveau, Koenig, & Harris, 2007; Sobel & Corriveau, 2010). Models of epistemic trust further suggest that preschoolers may be at a critical developmental point for default assumptions about adult knowledge states (Shafto, Eaves, Navarros, & Perfors, 2012). Second, there is a growing body of literature suggesting that social inferences are pervasive in children's reasoning about how observed physical data are generated (Bonawitz et al., 2011; Buchsbaum et al., 2011; Butler & Markman, 2012; Gweon, Tenenbaum, & Schulz, 2010; Horowitz & Frank, 2016; Kushnir, Xu, & Wellman, 2010).

The hypothesis we present here suggests that children do not simply use social inferences to enhance learning from observed, physical data. Rather, it suggests that children are prepared to learn from social situations, even in the absence of any direct evidence from the physical world. This provides an explanation for children's switching response to “neutral” follow‐up questions (they are a form of social data) and has powerful implications for theories of cognitive development, learning, and education.

The argument that meaning is in part determined by the choice of what not to say is related to ideas in the literature on pragmatics. For example, standard interpretations of scalar implicature suggest that adult use of the word “some” implies not all. Unlike the current situation, in a Gricean sense (Grice, 1975), these are explained through informativity; a speaker's choice of the word “some” would be underinformative if “all” were true. For children, the existence of such pragmatic implicature effects is less clear (Barner, Chow & Yang, 2009; Huang & Snedeker, 2009a, 2009b; Noveck, 2001; Papafragou & Musolino, 2003). Our approach differs in that we are asking whether underinformativity, together with past experience, guides interpretation of neutral (less informative) statement questions.

Our rational social inference hypothesis is also of practical significance. The prevalence of children's social inferences in neutral contexts would raise serious problems for how children's understanding is interpreted in a variety of situations. For example, in legal, educational, and experimental psychological settings, neutral questions are used to gauge the certainty of respondents in their answers. If, instead of serving as queries about children's certainty, repeated questions serve as a kind of social evidence that triggers belief updating, then we would have to reassess the use and validity of, as well as the answers obtained with, these techniques.

We explore this problem from computational, observational, and empirical approaches. Here we present a mathematical analysis of a learner's expectations following neutral follow‐up questions. We then assess the circumstances in which neutral queries are used in everyday parent–child speech from the CHILDES corpus, and relate these findings to our analysis. Following, we provide three experiments investigating the hypothesis that preschool‐aged children rationally update their beliefs in response to neutral follow‐up queries by knowledgeable and ignorant questioners. If children's tendency to switch responses following neutral questions simply reflects “noisy behavior” caused by methodological or cognitive limitations, then the knowledge state of the questioner should have no effect on children's responses. If, however, the rational social inference hypothesis is correct, then children should be more inclined to switch responses when queried by a knowledgeable experimenter. Taken together, our approach lets us explore the hypothesis that children are making a social inference when responding to otherwise “neutral” questions.

## 2 Computational framework: Assessing why and when a learner may switch beliefs following neutral queries

We implement a generative probabilistic framework to provide an explanatory account of why (and thus when) a learner would switch beliefs following a neutral question such as “is that your final guess” We consider the simple case where the child is asked a question and gives a response to which there may be one of three kinds of feedback from the questioner: “correct,” “incorrect,” or something neutral such as “is that your final guess” We ask, what should a child infer following each of these kinds of feedback? Hearing “correct” unambiguously lets the child know her initial statement is likely true; hearing “incorrect” unambiguously lets the child know her initial statement is likely false. However, “is that your final guess” is ambiguous. In fact, it may not only prompt a child to reconsider her initial guess, but lead the child to infer that her initial guess is incorrect given certain assumptions. Specifically, children might assume that adults have a “positivity bias”; adults might be inclined toward telling a child she is correct whenever appropriate but disinclined toward telling the child she is incorrect. If so, children might infer that adults provide neutral follow‐ups such as “is that your final guess” to give them a second chance (without negative feedback) at getting to the correct answer.

To make the discussion concrete and presage our experiments, we consider a specific example: guessing the location of a sticker under one of two cups. Children, without any information about the location, may be asked to guess which cup contains the sticker. An adult may then ask a neutral follow‐up question such as, “is that your final guess” The question is, should the child update her beliefs about the location of the sticker in response to this neutral follow‐up? In what follows, we formalize inference under different assumptions about the degree to which adults are inclined to give negative feedback.

Bayesian inference provides a natural framework in which to consider how an ideal learner should update her beliefs in this case. In Bayesian inference, the learner's goal is to update her beliefs about hypotheses, *h*, given data, *d*, where the degree of belief in a hypothesis given data is denoted *P*(*d*). These updated posterior beliefs are determined by two factors: the learner's prior beliefs in hypotheses, *P*(*h*), and the probability of sampling the observed data, assuming the hypothesis is true, *P*(*d*|*h*). Specifically, the updated posterior belief in a particular hypothesis is proportional to the product of the prior belief in that hypothesis and the probability of sampling the data given that hypothesis,

(1) $$P\left(h|d\right)\propto P\left(d|h\right)P\left(h\right).$$

Because we are considering only two hypotheses (about the location of the sticker), we can use Bayes Odds to simplify the problem:

(2) $$\frac{P(h_{1}|d)}{P(h_{2}|d)}=\frac{P\left(d|h_{1}\right)P\left(h_{1}\right)}{P\left(d|h_{2}\right)P\left(h_{2}\right)},$$

where *P*(*h*_1_ *d*) is the probability that the sticker is in the first location, given the statement from the informant (“correct,” “incorrect,” “are you sure”) and *P*(*h*_2_|*d*) is the probability that the sticker is in the second location given the statement. It is reasonable to assume that the learner has no a priori assumptions about either location, which means that the posterior depends entirely on the relative probability of the data under the two hypotheses (i.e., *P*(*h*_1_) = *P*(*h*_2_), thus $\frac{P\left(h_{1}\right)}{P\left(h_{2}\right)}=1$).

**Figure 1:**
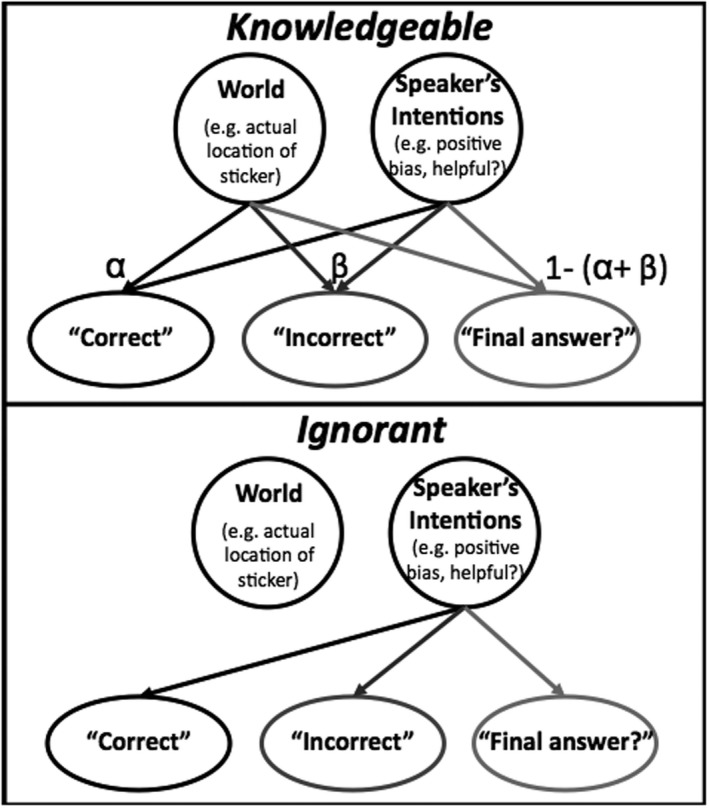
Graphical model depicting dependencies in cases when the informant is *knowledgeable* and *ignorant*.

Our analysis reveals that the main work comes down to the probability of the statement given the location of the sticker, *P*(*h*). We can model this likelihood with a simple causal graphical model (see Fig. 1). Causal graphical models consist of a structure indicating the causal relationships among a set of variables, where nodes are variables and dependence relationships are indicated by arrows from causes to effects. To complete a graphical model, conditional probability distributions give the probability that each variable takes on a particular value given the value of its causes (Pearl, 2000; Spirtes, Glymour, & Schienes, 1993) (Table 1).

**Table 1:** Conditional probability table for knowledgeable graphical model

Guess	“Correct”	“Incorrect”	“Are You Sure?”
Correct	α*_c_*	β***_c_*** ≈ 0	1 − (α*_c_ + *β**_c_**) ≈ 1 − α*_c_*
Incorrect	α*_i_* ≈ 0	β***_i_***	1 − (α*_i_ + *β***_i_***) ≈ 1 − β***_i_***

In our model of the problem, the variables include the actual state of the world (“World,” i.e., location of the sticker), the intention of the speaker (to provide helpful feedback, to avoid negative feedback, etc.), and the possible statements the speaker can make (“Correct,” “Incorrect,” “is that your final guess”). In the first model (*Knowledgeable*), the speaker or informant is aware of the actual state of the world. As a result, both the true location of the sticker and the intention of the informant influence the statement given to the learner. In the second model (*Ignorant*), the informant is not aware of the actual state of the world. As a result, the true state of the world does not influence the statement given to the learner. That is, the state of the world and the information provided are causally independent of each other.

The dependence assumptions captured by the graphical model generate predictions about the behavior of learners. In the case of a knowledgeable informant, given information about the actual state of the world and the informant's statement, a learner can infer something about the informant's goals (e.g., to avoid providing negative feedback, and to help the learner come to the correct answer). Given information about the state of the world and the informant's goals, a learner could also predict (with some probability) the likelihood that the informant would produce different statements. In our problem, given the informant's goals and the statement provided, the learner can make an inference about the state of the world. A learner could also make more abstract inferences: Given information about the goals of the informant, the statement provided, and the actual location of the sticker, a learner could infer which model (*Knowledgeable* or *Ignorant*) best captures the knowledge state of the informant.

The specification of the conditional probability distribution provides additional qualitative predictions. In the *Knowledgeable* graphical model, we might reasonably argue that if the child chooses the correct location initially, the speaker is very unlikely to say “incorrect”; in this case *P*(“incorrect” | correct choice) = β***_c_*** ≈ 0. Similarly, if the child chooses the incorrect location initially, the speaker is very unlikely to say “correct,” *P*(“correct” | incorrect choice) = α*_i_* ≈ 0. Given these intuitive assumptions, we can compare three possible ways that biases about the informant's goals might play out in the model's predictions.

The first possibility is that the informant is *unbiased*. Let us consider the case when the informant is knowledgeable. In this case, the *unbiased informant* is just as likely to say “correct” when the initial guess is correct as she is to say “incorrect" when the initial guess is incorrect, α*_c_* ≈ β***_i_***. If this is the case, then the learner cannot infer whether their initial guess is correct or not if they hear the statement “is that your final guess.” This is because *P*(“is that your final guess” | correct) = *P*(“is that your final guess” | incorrect). That is, the statement “is that your final guess” provides no additional information about the location of the sticker (Eq. 1 is approximately equal to 1).

Now consider the case where the informant is ignorant. In this case, because the informant has no information about the actual state of the world, the true location is conditionally independent of the statements made by the informant, and the learner cannot make any inferences about the state of the world. Thus, assuming unbiased informants, learners should make the same inferences if asked “is that your final guess” in a *Knowledgeable* condition as in an *Ignorant* condition.

A second possibility is that the informant is *positively biased*. In this model, the knowledgeable informant may be inclined to want to say “correct” following correct initial guesses, but would be reluctant to say “incorrect” following an incorrect initial guess, α*_c_* > β***_i_***. If this is the case, then the statement “is that your final guess” provides support for the hypothesis that the learner's initial guess was incorrect because she is more likely to hear “is that your final guess” given an incorrect guess than hear “is that your final guess” given a correct guess (Eq. 1 > 1). Thus, the *positively biased* model predicts that a learner should show increased switching in a *Knowledgeable* condition as compared to an *Ignorant* condition (in which the state of the world is still conditionally independent of the statements and thus does not provide additional information).

The third possibility is that the informant is *negatively biased*. In this model, the informant may be inclined to say “incorrect” following an incorrect initial guess, but would be comparatively reluctant to say “correct” following a correct initial guess, α*_c_* < β***_i_***. If this is the case, then the statement “is that your final guess” provides support for the hypothesis that the learner's initial guess was correct (Eq. 1 < 1), and the learner should show a decrease in switching responses in a *Knowledgeable* condition as compared to an *Ignorant* condition.

Note that the precise values of α and β are not important for the predictions of this model, but the relationship between these variables drives the predictive differences. We follow up on this model in three ways. First, we investigate whether neutral queries are used in everyday child‐directed speech and explore a possible positivity bias (a precondition of switching behavior) in these analyses. Second, we replicate the finding that preschoolers tend to switch responses following what might be considered a “neutral query” using a simple experimental paradigm. Third, this paradigm allows us to test a novel implication of our model—that preschoolers take the knowledge state of the informant into account when inferring whether or not to switch hypotheses.

## 3 CHILDES analysis: Neutral queries in everyday speech to children

To see whether this positivity bias is observed in naturalistic settings, we performed two analyses of the CHILDES database. The first involved a simple frequency analysis of the word pairs “right”/“wrong,” “correct”/“incorrect,” and “yes”/“no” from the CHILDES corpus (MacWhinney, 2000; for details of the analysis, see Appendix A and Table A1). We found that the “positive” responses outnumbered the negative responses by approximately 20 to 1. Although we cannot tell from this analysis how often children are correct and deserved positive feedback, it does suggest that children hear positive utterances more often than negative ones.

Our second analysis involved more thorough coding of parent–child conversations. First, we searched through the entire CHILDES corpus to identify transcripts that recorded day‐to‐day conversations at home between mothers and their young children between 2 and 6 years of age (details of the search criteria are listed in Appendix A). The search resulted in 166 transcripts from 24 studies. Two coders then identified the first 10 questions from mothers to children in these transcripts, and coded children's responses to these questions as well as mothers' possible follow‐ups. Children's answers were coded as irrelevant, relevant but not possible to judge correctness, correct, or incorrect. Contexts before and after the question were used to help determine the correctness of answers, and the two coders generally agreed on the correctness of answers (inter‐rater reliability was high: Cohen's κ = .85). Inconsistent codes were reviewed and resolved by a third coder.

For each answer, the two coders then coded if the mother followed up the child's answer with a positive response, negative response, or a neutral query. Positive response is defined as a statement that confirms the answer is correct, such as “Yes,” “Right,” “Good,” or repeating the answer as a statement (but not repeating the answer as a question). Negative response is defined as a statement that confirms the answer is incorrect, such as “No,” or “That's wrong.” Neutral query is defined as repeating the question or part of the question, or in rarer cases a statement or question that prompts children to answer the previous question again, such as “is that your final guess” or “Think about it.” Repeating children's answers in a question form or asking a clarification question (“What did you say?”) did not count as a neutral query. Inter‐rater reliability was high (Cohen's κ = .75), and inconsistent codes were reviewed and resolved by a third coder.

Of the 732 questions answered by children (i.e., their response was relevant), most resulted in a change of topic and did not receive a follow‐up response (506). Of those that received a follow‐up (226 coded questions), 144 (64%) received a positive follow‐up, 26 (12%) received a negative follow‐up, and 56 (25%) involved a neutral query follow‐up. These results reveal a tendency for parent–child conversations to involve questions that are answered and followed up with positive, more than negative feedback (**χ** ^2^(2) = 99.86, *p* < .001).

We also coded whether the child produced a correct or incorrect answer with respect to whether the parent was likely to follow up with a negative, neutral, or positive response. Of the 226 questions, coders were able to identify whether 84 responses were actually correct given questions that included independently verifiable statements of fact. Sixty‐four of these statements were correct, and of these 64, the vast majority were followed with a positive response (92%), and a small percentage were followed with a neutral query (8%). Of the 20 statements that were verified to be incorrect, most were followed by a negative response (65%), but a large percentage (35%) were followed by a neutral query. Thus, parents were significantly more likely to follow an incorrect statement with a neutral query than a correct statement, Fisher's exact test, *p* < .01).

These results suggest that parent–child conversations tend toward positive follow‐ups (often because parents appear to ask questions to which children know the answers), but also that neutral queries are commonplace in development, and these queries are more likely to follow incorrect responses than correct ones. Thus, this observation supports the model in which positive feedback is more common than negative feedback.

Importantly, in a world skewed toward positive feedback, rational learners *should* change answers in response to neutral questions: Neutral questions are significantly more likely to be generated following an incorrect response. However, our mathematical analysis predicts that this inference should apply when the questioner is assumed to be knowledgeable about the true answer. Neutral questions may also elicit some switching even when the questioner is ignorant, perhaps due to the child's uncertainty and because it might act as an invitation to reconsider an answer. Nonetheless, the rational social inference hypothesis predicts that the tendency to switch responses following a neutral query from a knowledgeable informant should be increased as compared to potential switching following a neutral query from an ignorant informant. In the remainder of the paper, we present empirical evidence that preschool‐aged children do switch responses following neutral queries, but do so in a manner that is sensitive to the knowledge state of the questioner, as predicted by the rational analysis.

## 4 Experiment 1: Responses to neutral queries following positive questioner training in Knowledgeable and Ignorant conditions

To investigate the predictions of our model, we assessed whether preschool‐aged children tend to switch responses following neutral questions in Knowledgeable and Ignorant conditions. We focused on preschool‐aged children because this age group has been reported to be particularly susceptible to switching responses after neutral questioning (Howie et al., 2004). Furthermore, past work on children's social inferences from physical evidence similarly shows that the inferences of children as young as 4 years are even sensitive to the knowledge and intentions of a speaker (Bonawitz et al., 2011), and models of children's epistemic trust suggest that it is around 4 years of age that children take an adult's knowledge state as a default (Shafto et al., 2012). However, we did not know what kinds of expectations children would have about the potential positivity of an unfamiliar questioner. Thus, to explore the increased switching behavior following neutral questions that is predicted by the positivity bias in our model, we first provided preschoolers with evidence that the experimenter tended to provide positive feedback to guessers. Then preschoolers were asked to participate by making guesses about the location of a sticker under one of two cups. In the critical test trial, the experimenter asked the child “is that your final guess” after children's first prediction. The critical measure was simply the proportion of children who switched their prediction to the other cup by condition.

### 4.1 Method

#### 4.1.1 Participants

Preschoolers were recruited from local preschools and an indoor play center in a diverse city community. Forty‐five children (*M*_age_ = 59.1 months; range = 51–70 months) were tested; one child was dropped in the Knowledgeable condition due to experimenter error in the training portion of the task,^1^ resulting in 22 children in the *Knowledgeable* Condition and 22 in the *Ignorant* Condition.

#### 4.1.2 Materials

In the positivity training portion, four cards were used. Each card had two cartoon pictures on it of items familiar to preschoolers (dog and cat; black cow and purple cow; small ball and large ball; star shape and square shape). In the Sticker task, five pairs of colored cups (pink, blue, yellow, green, and orange) were used. An animal sticker was placed on the inside of one of the cups in each pair.

#### 4.1.3 Procedure

Children were given two phases of the experiment, Positivity Training and Sticker Task.

##### Positivity training

Children first observed evidence that the experimenter tended to say “correct” to correct responses, and “are you sure” to incorrect responses.^2^ To show this, an adult confederate seated next to the child was presented with four cards by the experimenter, each card containing two pictures [dog vs. cat; black vs. purple cow; small vs. large ball; star vs. square shape]. The experimenter asked the confederate about each card [“Which is the dog? Can you point to the dog?” “Which is the purple cow, can you point to the purple cow?” etc.] On two of the four cards, the confederate made the correct choice clearly such that the observing child could see, and the experimenter said, “Look—he said that this one is the [dog; purple cow, etc.]. That's correct! So, can you {addressing the confederate} show me, which is the [dog; purple cow; etc.]?” As the experimenter asked this, she lifted the card so that it was pointing away from the child and so the confederate's follow‐up pointing final response could not be observed. On the other two cards, the confederate pointed to the incorrect response, and the experimenter said, “Look, he said this was the [larger ball; star; etc.]. Is that your final guess {addressing the confederate}? So can you show me, which is the [larger ball; star; etc.]?” The full statement was provided without pause or interruption from the confederate. As with the positive card conditions, the final point was obscured from the child's view.

##### Sticker task

*Initial phase*. The experimenter began by pulling aside a pair of cups and showing the child that there was a sticker inside one and no sticker inside the other. The experimenter then said, “In this game, it is going to be your job to guess which cup has the sticker inside. For each set of cups I'm going to ask you twice which cup you think has the sticker inside. After you make your first guess I will ask you once more and you can either keep your guess the same or guess the other cup. If your second guess is right, then you get a point and for every point you get we will play a game at the end. So remember you want to try and get your second guess right so you can get a point!” The experimenter then proceeded to take a different pair of cups and said, “Let's take a look at these two cups. One of them has the sticker inside and it's going to be your job to guess which one. I'm going to look inside so I know which cup has the sticker.” The experimenter looked inside and then asked the child which cup (s)he believed had the sticker. Regardless of the accuracy of the guess, the experimenter responded, “Yes that's right!” and then asked the child again which cup (s)he believed had the sticker inside. Children did not see the contents of the cup immediately after the trials, so they did not receive feedback as to whether their final guesses led to the selection of the cup with the sticker inside. The experimenter repeated this procedure, saying “yes that's right” after the first guess on a second, new pair of cups.

**Figure 2:**
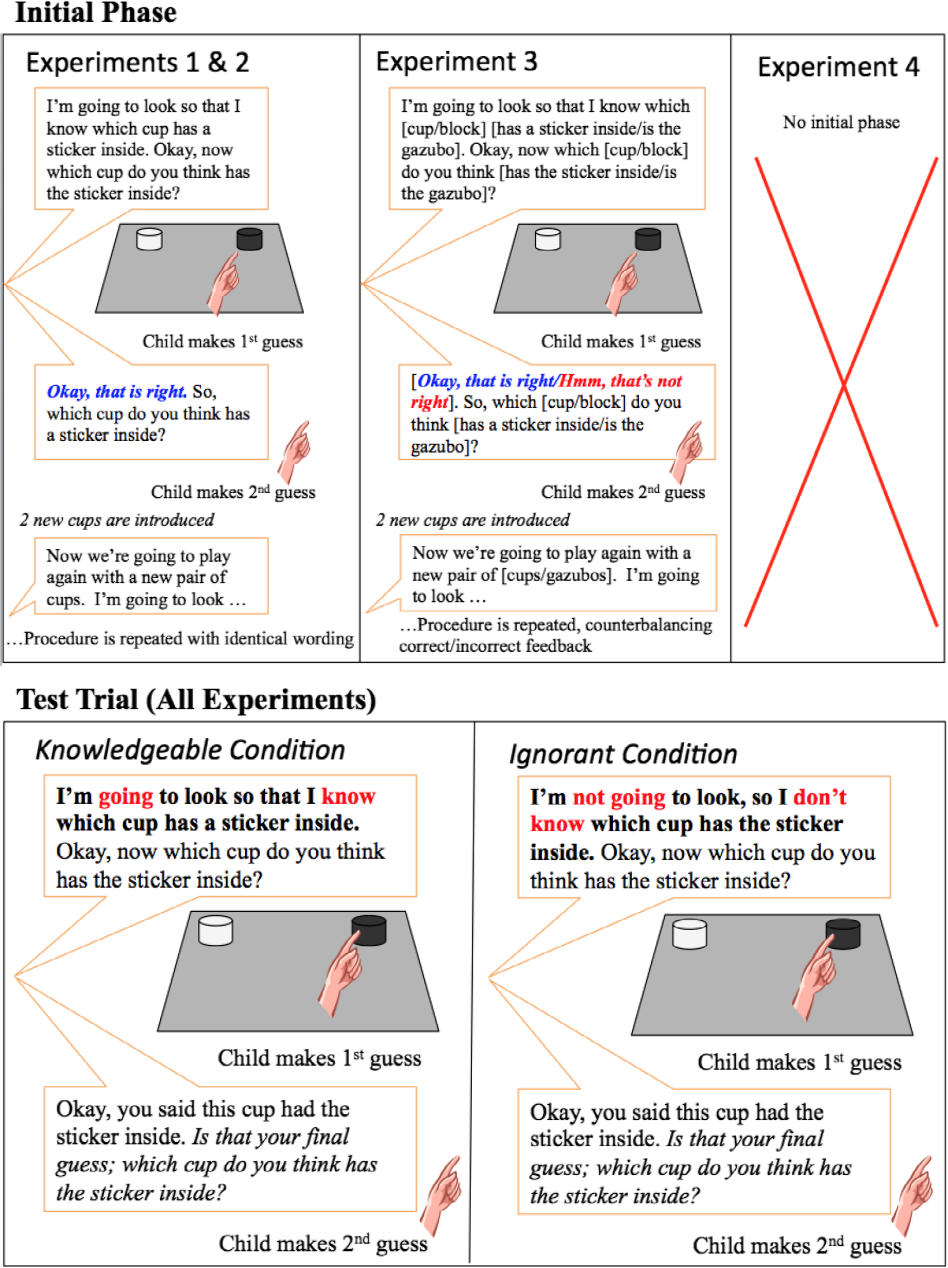
Procedure for the sticker [gazoob] task for Experiments 1–4, including initial phase and test trial of the *Knowledgeable* and *Ignorant* conditions.

*Test trial*. (See Fig. 2.) Following the two “correct” feedback trials, the experimenter brought out a third and final pair of cups. In the *Knowledgeable* condition the experimenter said, “I'm going to look so I know which cup has the sticker inside,” making her knowledge state explicit. She then proceeded to ask the child which cup had the sticker inside; when the child responded, the experimenter provided no explicit feedback as in the preceding confirmation trials, but instead said, “Okay, you said this cup had the sticker inside. Is that your final guess; which cup do you think has the sticker inside?” Children did not see the location of the sticker. In the *Ignorant* condition the experimenter said, “This time, I'm not going to look inside so I don't know which cup has the sticker either,” making her ignorance explicit; the rest of the *test trial* proceeded in the same way as in the *Knowledgeable* condition. Experimenters were trained to use a neutral tone for the follow‐up “Is that your final guess” prompt, to ensure no social cue or prosody differences were accidentally generated between conditions. A subset of clips were coded by a research assistant blind to condition, confirming that there were no vocal or social cues that could be used to distinguish the prompt in the two conditions. At the end of the experiment, the experimenter brought back all the pairs of cups and let the children discover which cups contained the stickers (see Fig. 2).

### 4.2 Results and discussion

**Figure 3:**
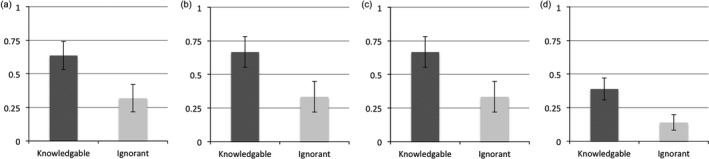
Children in Experiment 1s (a), 2 (b), 3 (c), and 4 (d) were more likely to switch responses following a neutral query from a knowledgeable experimenter than from an ignorant one (error bars represent *SE* calculated by dividing the *SD* by the square root of the sample size as discussed in Brown, Cai, & DasGupta, 2001).

Children's final responses were coded live by the experimenter and corroborated by the observing confederate. We predicted that children in the *Knowledgeable* condition would switch their guesses more than children in the *Ignorant* condition. To test this prediction, we compared the frequency of switching and staying across the two conditions. Our results revealed that children were more likely to switch in the *Knowledgeable condition* (14/22) than in the *Ignorant condition* (7/22), Fisher exact test,^3^ switched, *p* = .034, effect size as odds ratio = 3.75 (see Fig. 3a). The different pattern of responses between our conditions suggests that children are sensitive to the epistemic state of questioners when deciding how to respond to neutral follow‐up questions.

We noticed that a few children tended to pick a side (e.g., the right side) on the training trials, and then stuck with it throughout the experiment. This may have happened because the child received confirming information (“that's right”) on the initial training trials and so may have inferred a rule that the sticker would always be on the one side. Although it is difficult to ascertain from only three trials, we return to and address this point in Experiments 2 and 3, with the addition of methodological check questions.

Results from our experiment corroborate past results that children switch responses following neutral queries. However, as predicted by our model, children do not do so indiscriminately. When children are introduced to an experimenter with a positivity bias, children were more likely to switch responses when the questioner was knowledgeable about the correct answer than when she was not, suggesting that children's switching behavior can be rationally explained by accounts appealing to the epistemic state and intentions of the questioner.

While this first study provides initial evidence supporting our claim, we were interested in replicating this effect. Furthermore, while children in Experiment 1 were given the opportunity to learn that the experimenter had a positivity bias, our CHILDES analysis suggests that this bias may already be in place (at least with parent–child dyads). One possibility is that this positivity bias extends to all adult questioners and does not require initial exposure. We tested this possibility in Experiment 2.

## 5 Experiment 2

In Experiment 2 we sought to replicate Experiment 1, with the change that children were not given information about the response biases of the questioner prior to the sticker task. We also included a methodological check that would allow us to identify children who accidentally inferred a “side rule” due to the nature of the feedback in the initial phase of the experiment.

### 5.1 Method

#### 5.1.1 Participants

Preschoolers were recruited from local preschools and science museums near a university campus in an urban area. Thirty‐six preschoolers (*M*_age_ = 59 months; range = 48–72 months) were tested and randomly assigned to either the *Knowledgeable* or *Ignorant* condition. An additional 10 children were replaced for following a “side rule,” exhibiting a same‐side pattern of responding throughout the initial phase, test, and methodological check trials as explained below.

#### 5.1.2 Materials

The same five pairs of colored cups (pink, blue, yellow, green, and orange) and stickers were used as in Experiment 1.

#### 5.1.3 Procedure

The experiment proceeded identically to Experiment 1, with the exception that we did not include the initial positivity task. Thus, the experimenter began with pulling aside a pair of cups and showing the child that there was a sticker inside one and no sticker inside the other as in the Sticker Task in Experiment 1. Regardless of the accuracy of the children's guess, the experimenter responded, “Yes that's right!” and then asked the child again which cup (s)he believed had the sticker inside. This training trial was repeated a second time with a different pair of cups.

The test trial then also proceeded identically as in Experiment 1, with half the children observing a knowledgeable experimenter, who looked, and half observing the ignorant experimenter, who did not look (see Fig. 2).

Following the test trials, we included two additional methodological check trials, which allowed us to assess whether the child exhibited having inferred a side rule. We provided children with a new Sticker task on these final two trials and coded which sides children selected.

#### 5.1.4 Coding

Children's responses were videotaped and coded by an assistant blind to condition; seven children were coded live because either no video consent was provided by the parents or because the view of the children's pointing response was obstructed. For the remaining 25 children, there was 92% agreement; the errors were caused by obvious Left/Right coding errors and were resolved by a third coder.

Coders also identified children who needed to be dropped for inferring a side rule. They identified whether the participant chose the same side in the first two training trials, which was followed by the confirming feedback, and then whether children continued to choose this side in the test trial and methodological check trials. A child was confirmed to have inferred a side rule if she chose the cup on that same side for the test and method trials, following evidence that that side contained the sticker in at least one of the training trials. Ten children (4 in the Knowledgeable condition and 6 in the Ignorant condition) were dropped and replaced based on these criteria.

### 5.2 Results and discussion

We first confirmed that children interpreted the positive “yes” response feedback in the initial trials as indicating a correct answer. Of the 72 trials (2 trials for each of the 36 participants), every single child stayed with their initial answer when provided positive feedback. This confirmed that children understood the task and were motivated to provide a correct response.

For our primary measure of interest, we predicted that children in the *Knowledgeable* condition would switch their guesses more often than children in the *Ignorant* condition (see Fig. 3b). The frequency of switching and staying across the two conditions again differed, with children in the *Knowledgeable condition* switching (12/18) more than children in the *Ignorant condition* (6/18), Fisher exact test, *p* = .047; effect size as odds ratio = 4.0.^4^ These results add further support to the claim that children are sensitive to the epistemic state of questioners when deciding how to respond to neutral follow‐up questions.

In the training, children were given positive feedback in both trials. Although repeated positive feedback does not predict the difference we observed between conditions, one could argue that the training trials may have influenced children's assumptions about the relative frequency of positive feedback, which may have augmented the proportion of switching in the knowledgeable condition. To address this question, we conducted a third experiment in which the training contained one trial with positive feedback (as above) and one trial with negative feedback. We also included a new causal task to assess the generalizability of the phenomenon.

## 6 Experiment 3

In our third experiment, we balanced the positive and negative information about the objects prior to the critical test trial. We were also interested in generalizability across domains, so we included both the original sticker task and also a new causal inference task, in which children guessed which of two identical blocks was the block that would make a machine light up and play music. As with the sticker task, children did not observe the outcomes of their guesses until the very end of the experiment. Finally, we included the methodological side rule check as in Experiment 2.

### 6.1 Method

#### 6.1.1 Participants

Preschoolers were recruited from local preschools and museums. Participants were randomly assigned to the Sticker Task *Knowledgeable* or *Ignorant* conditions (*n* = 18 per condition, *M*_age_ = 58 months, range = 48–80 months) or to the Causal Inference Task *Knowledgeable* or *Ignorant* conditions (*n* = 18 per condition, *M*_age_ = 61 months, range = 49–71 months). As with Experiment 2, we coded children for a side rule induced by the initial feedback using a final two catch trials to determine dropping criteria; an additional 16 children (8 in *Knowledgeable* and 8 in *Ignorant*) were dropped based on these criteria. This resulted in 72 children as the final analyzed total with equal numbers across task type and condition.

#### 6.1.2 Materials

##### Sticker tasks

The same materials were used as in Experiments 1 and 2.

##### Causal task

Materials included five pairs of orange blocks and a two‐tiered cardboard box covered in felt with the top tier acting as an “activator.” A children's fan toy that lights up and spins when a button is pressed was affixed to the top of the box with the button hidden from the child's view.

#### 6.1.3 Procedure

##### Sticker task

The sticker task used in Experiment 3 was the same as the Sticker task in Experiments 1 and 2 except that during the training trials, children received positive feedback (“Yes that's right!”) after their first guess for one trial and negative feedback (“Hmm that's not right”) after their first guess for the other trial. Whether or not they received positive feedback first was randomized across participants (see Fig. 2).

##### Causal task

The experimenter began by pulling aside a pair of identical blocks (referenced as toys) and explaining to the child that one of the blocks was called a “gazubo” and one of the blocks was not. The experimenter then said, “In this game, it is going to be your job to guess which of my toys are gazubos. See my machine here? Well, when we put certain kinds of toys called gazubos on it, my machine lights up and spins.” The experimenter then took one block and placed it on the machine and said, “This toy made my machine light up and spin, is it a gazubo?” After the child answered, the experimenter either confirmed or corrected the child's answer and then took the other block and placed it on the machine and asked, “This toy did not make my machine light up and spin, is it a gazubo?” again correcting or confirming the child's answer. The experimenter then began explaining the task, “We're going to play a game where your job is to guess which of my two toys is a gazubo. After you make a guess, I'm going to ask you one more time which toy you think is the gazubo and you can either keep your guess the same or guess the other toy. If your second guess is right, then you get a point and at the end we'll play a game for every point you have. So you want to try to get your second guess right, because otherwise you won't get a point!”

The experimenter then proceeded to take out a different pair of blocks and said, “Let's take a look at these two toys. One of them is a gazubo and the other is not. I've already played with these toys so I know which one is a gazubo.” The experimenter then asked the child which block they believed to be the gazubo. Regardless of the accuracy of the child's guess, the experimenter randomly responded either “Yes, that's right!” or “Hmm, that's not right” and asked the child again which block they believed to be the gazubo. Children did not see the block placed on the machine after the trial, so they did not receive feedback as to whether their guesses were correct. The second training trial was the same as the first with the exception that the experimenter reversed the response provided after the child's initial guess.

The experimenter then began the test trials. In the *Knowledgeable* condition the experimenter said, “I've already played with these toys so I know which one is a gazubo” making her knowledge state explicit. She then proceeded to ask the child which block (s)he believed to be the gazubo; when the child responded, the experimenter provided no explicit feedback as in the training trials, but instead said, “Okay, you said this toy was the gazubo. Is that your final guess; which toy do you think is the gazubo?” Children did not see the block placed onto the machine. In the *Ignorant* condition the experimenter said, “I haven't played with these toys before, so I don't know which one is the gazubo either” making her ignorance explicit; the rest of the test trials proceeded as with the *Knowledgeable* condition. At the end of the experiment, the experimenter brought back all the pairs of blocks and let the children discover which ones activated the machine.

##### Coding

Children's responses were videotaped and coded by an assistant blind to condition. A portion of responses (40%) were reliability coded; there was 100% agreement.

### 6.2 Results and discussion

There were no differences between the Sticker and Causal tasks by condition (*Knowledgeable*: *p* = 1; *Ignorant*: *p* = 1 by two‐tailed Fisher's exact test), so we collapsed the data across tasks. We first assessed whether children understood the initial phase of the task—that they should stay with their correct response if provided positive feedback and switch if provided negative feedback. Children overwhelmingly stayed with their responses when told “yes” after their guesses in the initial phase (66/72 children) and overwhelmingly switched responses when told “no” (68/72).

As with Experiments 1 and 2, our primary question was whether children were sensitive to the knowledge state of the experimenter when reasoning from neutral feedback in the test trials. Replicating these first two experiments, children switched more in the *Knowledgeable* condition (22/36) than in the *Ignorant* condition (13/36), Fisher exact test, *p* = .034; effect size as odds ratio = 2.78^5^; See Fig. 3c. This result replicates Experiments 1 and 2 and provides further evidence that children are sensitive to the epistemic state of questioners when answering follow‐up questions on a variety of tasks.

## 7 Experiment 4

Experiments 1–3 provided consistent evidence that children are sensitive to the epistemic state of a questioner and use this information to guide interpretation of pragmatically ambiguous statements, leading to more switching responses following otherwise neutral follow‐ups. All experimental conditions started with a knowledgeable experimenter in the initial phase trials, but this state switched for the critical test trial in the *Ignorant* (Experimenter) conditions. Thus, one deflationary account to our results is that something about moving from a knowledgeable to ignorant state differently affected children in the *Ignorant* conditions, leading to different patterns of responding between conditions independent of the knowledgeability hypothesis. In Experiment 4 we explored this deflationary account by removing all initial phase trials, so as not to induce any expectations for participants.^6^

### 7.1 Method

#### 7.1.1 Participants

Preschoolers were recruited from local preschools near a university campus in an urban area. Seventy‐two preschoolers (*M*_age_ = 56.2 months; range = 48–71 months) were tested and randomly assigned to either the *Knowledgeable* or *Ignorant* condition. No children were dropped and none excluded for a “side rule.”

#### 7.1.2 Materials

One pair of red cups and stickers were used, as in Experiment 1.

#### 7.1.3 Procedure

The experiment proceeded identically to Experiments 1 and 2, with the exception that we did not include any initial sticker phase. Thus, the experiment only involved the single test phase (see Fig. 2).

#### 7.1.4 Coding

Children's responses were videotaped and coded by an assistant during testing; another assistant who was blind to condition checked all responses from video (reliability = 100%). Because there were no training trials (just the single test trial), there was no way for children to learn or exhibit a side rule, and so this additional check was not included.

### 7.2 Results and discussion

We predicted that children in the *Knowledgeable* condition would switch their guesses more often than children in the *Ignorant* condition. Fig. 3d shows the results. The frequency of switching and staying across the two conditions again differed, with children in the *Knowledgeable* condition switching (14/36) more than children in the *Ignorant* condition (5/36), Fisher exact test, *p* = .015; effect size as odds ratio = 3.95. These results help to rule out the alternative explanation that it was simply changing the knowledge state in the *Ignorant* condition—but not the ignorance per se—that led to the observed differences in Experiments 1–3. Here there were no preceding trials, so children could only rely on whether the experimenter was knowledgeable or not to guide their response behavior. These results replicate the previous Experiments 1–3, with children switching more in *Knowledgeable* conditions.

## 8 General “mega‐analysis” results

Recapitulating the main findings of Experiments 1–4, across all studies, children switched more in the *Knowledgeable* condition (62/112) than in the *Ignorant* condition (31/112), Fisher exact test, *p* < .0001 (effect size as odds ratio = 3.24). Comparing across Experiments 1–3 revealed no differences in the total amount of switching among *Knowledgeable* conditions (χ^2^ = .16, *p* = .923) and no differences among *Ignorant* conditions (χ^2^ = .12, *p* = .942); however, there was a significant difference between Experiment 4 and these three experiments, with children switching less overall in both the *Knowledgeable* (χ^2^ = 5.82, *p* = .015, effect size as odds ratio = .37) and *Ignorant* conditions (χ^2^ = 5.04, *p* = .025, effect size as odds ratio = .31).

We explored whether children's switching behavior was different than chance responding would predict (i.e., if children guessed at chance every time they responded to a question, then the probability of switching between guess 1 and 2 is .5). Due to the overall switching differences between Experiment 4 and Experiments 1–3, we analyzed these results separately. First, we collapsed the results from Experiments 1–3 and compared the frequencies of switching for *Knowledgeable* and *Ignorant* conditions to chance performance. Although our model predicts overall differences between *Knowledgeable* and *Ignorant* conditions, we do not have a prediction for whether the total switching in each of these conditions should fall above, at, or below chance. Thus, we report two‐tail *p* values. Across Experiments 1–3, the switching in the *Knowledgeable* condition (48/76) was significantly greater than chance (binomial, *p* = .029), whereas the switching in the *Ignorant* condition (26/76) was significantly lower than chance (binomial, *p* = .008). However, the switching in the *Knowledgeable* condition of Experiment 4 was not significantly different from chance (binomial, *p* = .243), although switching in the *Ignorant* condition of Experiment 4 was again significantly below chance (binomial, *p* < .0001). Taken together, the results show that when an experimenter is known to be ignorant, children tend to stick with their initial responses following neutral questions. However, under some conditions (perhaps with greater experience with a positive experimenter, or following a brief warm‐up, as experienced in Experiments 1–3), children are more likely to change their minds in response to neutral queries.

We also explored whether switching behavior changed with age. By median age split, overall older children (55/112) were more likely to switch than younger (38/112), (χ^2^ = 5.31, *p* = .021, effect size as odds ratio = .53), but this was primarily driven by a marginal effect of more switching by older children in the *Knowledgeable* conditions (36/56) than younger children (26/56), (χ^2^ = 3.61, *p* = .057, effect size as odds ratio = .48); there were no significant differences in switching in the *Ignorant* conditions between older (18/56) and younger (13/56) children (χ^2^ = 1.12, *p* = .290, effect size as odds ratio = .64). That is, as children were older in the *Knowledgeable* conditions, the more likely they were to switch, *r*(110) = .19, *t* = 2.01, *p* = .047, but this was not the case in the *Ignorant* conditions, *r*(110) = .001, *t* = −0.34, *p* = .735. These results suggest that there may be a developmental effect of sensitivity to knowledgeability leading to switching responses following neutral questions. Slightly older preschoolers may be more sensitive to the pragmatic implication of such a follow‐up and may thus be more likely to revise their responses.

## 9 General discussion

Children often change responses to seemingly neutral questions, which has been interpreted as a sign of children's uncertainty and limited memory capacity. We proposed an alternative explanation for switching behavior by introducing a causal model of response evaluation that included the questioner's knowledge state as a critical component of children's assessment of their own answers. This model predicted different interpretations of neutral queries based on the underlying assumptions of the questioner—whether the questioner is assumed to be positive/helpful, neutral, or negative/unhelpful. Greater switching behavior is predicted by a positivity bias, and initial corpora analyses suggest that children's conversational environments tilt toward this positivity bias. Our four follow‐up experiments find that children switch more often to neutral questions from a knowledgeable questioner, which further confirms this positivity bias. Importantly, as predicted by our model, children take the knowledge state of the questioner into account; children are more likely to switch following a neutral question from a knowledgeable questioner than from an ignorant one.

These results suggest that by at least the preschool years, children are prepared to learn from social situations. Specifically, seemingly neutral follow‐up questions may lead children to change their minds. Children's switching is not simply triggered by any follow‐up query: When asked by a knowledgeable adult, children were more inclined to change their response than when asked by an ignorant adult. We predicted this result based on rational social inference: When people are more likely to give positive feedback for correct responses than negative feedback for incorrect responses, neutral follow‐up questions should indicate to the learner that she should update her beliefs. However, this assumption only holds if the questioner is knowledgeable. There is theoretically no additional evidence about the accuracy of an initial guess when the questioner does not have knowledge of the correct answer.

While our model predicted differences between knowledgeable and ignorant conditions, we did not have strong predictions regarding whether either condition would produce switching behavior significantly greater than or less than chance. Across Experiments 1–3, we found that, while children in the *Ignorant condition* were unlikely to switch responses (they were significantly less likely than chance to do so), children did switch more than chance responding would predict in the *Knowledgeable condition*. This suggests that children are not simply confused by repeated questioning, leading to chance responding. Children may be predisposed to stick with their gut response under uncertainty, but they may use information from the environment (e.g., the social cues of others) to revise responses when it is available.

However, this switching‐above‐chance result did not hold for Experiment 4. Although children in this condition were significantly more likely to switch following a neutral follow‐up from a knowledgeable experimenter, they were not significantly more likely to switch in this condition as compared to chance. One possibility for the difference between Experiments 1–3 and Experiment 4 could be that children were more uncertain about the positive nature of the questioner in Experiment 4 because they had no experience in warm‐up trials or initial practice trials. However, the overall effect size was similar to that in Experiments 1–3, suggesting that the positivity bias was equally effective. The difference was merely a shift to less switching across both conditions of Experiment 4. Keep in mind that there is an inherent ambiguity in a follow‐up question: The askee does not know whether they are being re‐questioned because the initial answer was not heard or because the questioner is signaling that the response should be changed. Children in Experiments 1–3 received initial trials in which the experimenter practiced giving follow‐up questions to the child, setting the expectation that follow‐ups were the norm (and not due to an unheard response), but children in Experiment 4 did not receive this information. Thus, they may have been less certain whether the follow‐up was due to the questioner not hearing the answer, in which case the rational response is to stick with, rather than switch, responses.

One might question the generalizability of these findings, as we chose simple, relatively novel situations. Of course, we purposefully created a context in which children would have uncertainty about the correct answer. After all, if children had higher confidence in their beliefs, they would likely rely less on ambiguous social cues to inform their decision. This might occur because children are more likely to reference adults given uncertainty (Hembacher & Frank, 2017) or because additional information from another would hold less weight as compared to children's own knowledge (Bridgers et al., 2016). Such sensitivity implies that at some level children are aware of their own uncertainties, but that they help to resolve these uncertainties by depending on others.

While the scenarios we employed for our methods do not capture the full richness and context of children's everyday experiences, they do demonstrate the potential pervasiveness of children's inferences from social feedback. There is no direct reason why, across relatively novel tasks and multiple different experimenters, children should assume that the positivity bias and its logical implications hold. Nonetheless, that we obtain these results even in conditions such as Experiment 4, where very little information is known about the experimenter, implies that children are leveraging prior experiences across situations and people. Furthermore, our mega‐analysis suggests this ability may get stronger with development in the preschool years. That children use prior experience to guide inference should not be surprising; myriad evidence supports this point (Bonawitz et al., 2012; Schulz, Bonawitz, & Griffiths, 2007; Schulz, Gopnik, & Glymour, 2007). What is surprising is that experience is used to impute information about social goals from seemingly neutral, uninformative acts. Indeed, recent theory has argued that reasoning about people and why and whether they choose to act is a central feature of human cognition that supports development and explains cultural evolution (Tomasello et al., 2005). Such a claim suggests that we should carefully consider how we interpret children's actions, inferences, and responses when interacting with presumably knowledgeable adults.

Our results raise important practical considerations in legal, educational, and standard experimental settings. Oftentimes lawyers, teachers, and scientists ask children questions as a means of evaluating what the child believes. These questions are assumed to be inert, providing no additional information to the child, and the responses are treated as windows into the child's thinking. For example, in legal research practice, studies have shown that up to a third of jurors agree with the statement that “inconsistencies in a child's report … indicate that the report is false” (Quas, Thompson, Alison, & Stewart, 2005). Our results call both of these assumptions into question. Even seemingly neutral queries provide information to children when posed by someone who can reasonably be assumed to be knowledgeable. And, because children are savvy social operators, they may change their minds in response to these neutral queries. In legal settings, approaches to minimize repeated questioning across multiple interviews are often followed, focusing on open‐ended prompts (e.g., see La Rooy, 2010), but without a clear basis for when and how direct follow‐ups could be more reliably employed when needed. In educational settings, teachers might consider clarifying the pedagogical purposes of questioning and being explicit about their neutral intent. In research, methods should avoid redundant follow‐ups if possible, and researchers should be careful to note when follow‐ups lead to shifting responses.

Our approach involved pairing information about the statistics of children's actual experience, as documented in the CHILDES database, with computational models, and experimental methods. This supports the generalizability of our findings. We documented phenomena that are observed in experience, we analyzed their implications for behavior, and we confirmed these predictions in a controlled experiment. We conclude that children use prior experience to support inferences about what they should believe even when provided with information intended to be neutral by adults. Our approach does not exclude the possibility that other factors influence real‐world responses, but it does provide a strong case that the phenomenon exists and that it can and likely does play a role in children's behavior.

Our approach also suggests the kinds of information we would need in order to investigate the precise implications for legal, educational, and experimental contexts. CHILDES provides a rich source of information about children's typical experiences.^7^ This information is invaluable for constraining computational models and assessing the potential generality of experimental results. Thus, it is important that we collect and curate such observational data about the kinds of situations to which we wish to generalize.

In summary, by pooling the strength of observational data, computational models, and experimental methods, we have shown that seemingly neutral questions, such as “is that your final guess” when posed by knowledgeable adults, function as cues for children to update their beliefs. We have shown this is a rational inference based on children's actual experiences: Children are more likely to be told that they are correct when they are in fact correct, than they are to be told they are incorrect when they are incorrect. Assuming children track these experiences, neutral responses by a knowledgeable adult imply that they should change their beliefs. It is important for future work to assess the degree to which these findings impact practical legal, educational, and experimental contexts in which neutral queries are commonly used and interpreted as measures of children's certainty. Instead of offering a passive window into children's actual thoughts, these “neutral” queries may be actively changing what children think.

## Notes

1 Child responded in the direction of the predicted results and does not affect analyses.

2 We also piloted a version of the study whereby the experimenter was “negative” and offered “Are you sure” to correct responses and “No, that's wrong” to incorrect ones. We terminated this condition when it became clear that children were somewhat uncomfortable with the negative experimenter and were hesitant to participate in the remainder of the study with her.

3 Due to a priori predictions for directional differences between conditions, we report one‐tailed tests for Fisher exact test throughout.

4 Including the children who demonstrated a side‐bias does not change this result: Children in the Knowledgeable condition switched (12/21) more than children in the Ignorant condition (7/25), Fisher exact test, *p* = .044.

5 Including the children who demonstrated a side‐rule does not change this result: Children in the *Knowledgeable condition* switched (22/44) more than children in the Ignorant condition (13/44), Fisher exact test, *p* = .040.

6 We thank Amy Perfors for bringing this alternative to our attention and for this experimental suggestion.

7 It should be noted that though CHILDES provides a rich source for day‐to‐day parent–child conversations, the samples included in the corpora may not be representative. For example, it is known that children from highly educated middle‐class families are overrepresented in the database (Diessel, 2009).

## Appendix A CHILDES searches

For the first analysis, we used the CLAN commands available to the CHILDES database to estimate the frequencies of the word pairs “right”/“wrong,” “correct”/“incorrect,” and “yes”/“no” in the CHILDES corpora.

For the second, more comprehensive analysis, we searched through the CHILDES corpus to identify transcripts that recorded day‐to‐day conversations at home. A preliminary survey of the CHILDES database showed that 99% of the transcribed naturalistic family conversations had the mother present, whereas only 37% had the father present. Therefore, we focused on mother–child conversations to maximize usable transcripts. We searched the entire database for transcripts that meet the following eight criteria:

1. The transcript was in English.
2. The conversation took place at home.
3. The conversation partners included a mother and a child, and did not include anyone outside the immediate family (interviewer, grandparents, friends, etc.).
4. The target child was between 2 and 6 years of age.
5. The conversation represented everyday talk and was not a purposeful conversation such as an interview.
6. The transcripts for mother's and child's speech were not separated.
7. The transcript used punctuation marks, and it had at least three question marks in it.
8. If there were multiple transcripts for a same child (such as in longitudinal studies), we only used the first (earliest) transcript that meets all other criteria.

**Table A1:** Descriptions of Transcripts Used in the Study

Original study	# of trnscrpts. included	Era of data collected	Location of data collected	Child's age (mos)	Child's sex	Family SES
Bohannon III and Marquis (1977) and Stine and Bohannon (1983)	1	70s	USA	36	M	NS
Braunwald (1976)	1	70s	USA	37	F	NS
Demetras (1986, 1989)	3	80s	USA	25‐26	3M	NS
Demetras, Post, and Snow (1986) and Post (1994)	2	80s	USA	24‐31	2F	Working‐class
Demuth, Culbertson, and Alter (2006)	6	00s	USA	24‐27	3M 3F	NS
Dickinson and Tabors (2001)	58	80s	USA	43‐68	30M 28F	Low‐income
Feldman and Menn (2003)	1	90s	USA	25	M	NS
Gleason (1980)	13	70s	USA	32‐62	6M 7F	NS
Hayes and Ahrens (1988)	2	80s	USA	36‐40	1M 1F	Working‐class
Henry (1995) and Wilson and Henry (1998)	3	90s	UK	33‐54	2M 1F	NS
Howe (1981)	12	70s	UK	24	7M 2F	Specified for each family
Kuczaj (1977)	1	70s	USA	36	M	NS
Lieven, Salomo, and Tomasello (2009)	1	00s	UK	36	M	Middle‐class
McCune and Vihman (1987)	4	80s	USA	24	2M 2F	NS
Rowland and Fletcher (2006)	1	90s	UK	36	F	NS
Sachs (1983)	1	70s	USA	39	F	NS
Snow (1983)	1	70s	USA	30	M	NS
Suppes (1974)	1	70s	USA	36	F	NS
Theakston, Lieven, Pine, and Rowland (2001)	10	90s	UK	24‐32	5M 5F	NS
Van Houten (1986)	20	80s	USA	28	13M 7F	NS
Warren‐Leubecker and Bohannon III (1984)	11	80s	USA	24‐70	6M 5F	Middle‐class
Weist, Pawlak, and Hoffman (2009) and Weist and Zevenbergen (2008)	1	00s	USA	38	M	Middle‐class
Wells (1981)	11	70s	UK	26‐56	4M 7F	NS
Unpublished research by Julie McMillan	1	00s	USA	28	F	NS

Note.

NS, not specified; SES, socioeconomic status.

**Examples of transcripts**

**Table:**

Correct answer + neutral query:			Incorrect answer + neutral query:		
18	*MOT:	when you went to the airport with aunt Julie?	15	*MOT:	what color is that?
19	*MOT:	what'd ya'll see?	16	*CHI:	dat's [: that's] a w@l
20	*CHI:	airplane	17	*MOT:	what color?
21	*MOT:	you did?	18	*CHI:	w@l!
22	*CHI:	&uh huh	19	*MOT:	what color?
			20	*CHI:	what's dat [: that]?
			21	*MOT:	you're not listening
			22	*MOT:	what color is this?
			23	*CHI:	that's a puzzle
			24	*MOT:	I know it's a puzzle, but what color is it?
			25	*CHI:	is a puzzle
